# Influence of contemporary CAD-CAM milling systems on the fit and adaptation of partially stabilized Zirconia fixed partial dentures

**DOI:** 10.12669/pjms.37.1.3490

**Published:** 2021

**Authors:** Khulud A Al-Aali, Rana S Alhamdan, Ahmed M Maawadh, Fahim Vohra, Tariq Abduljabbar

**Affiliations:** 1 Khulud A Al-Aali Department of Clinical Dental Sciences, College of Dentistry, Princess Nourah Bint Abdulrahman University, Riyadh, Saudi Arabia; 2 Rana S Alhamdan Department of Restorative Dental Sciences, College of Dentistry, King Saud University; Riyadh 11545, Saudi Arabia; 3 Ahmed M Maawadh Department of Restorative Dental Sciences, College of Dentistry, King Saud University; Riyadh 11545, Saudi Arabia; 4 Fahim Vohra Department of Prosthetic Dental Science, College of Dentistry, King Saud University, Riyadh 11545, Saudi Arabia. Research Chair for Biological Research in Dental Health, King Saud University, Riyadh, Saudi Arabia; 5 Tariq Abduljabbar Department of Prosthetic Dental Science, College of Dentistry, King Saud University, Riyadh 11545, Saudi Arabia. Research Chair for Biological Research in Dental Health, King Saud University, Riyadh, Saudi Arabia

**Keywords:** Marginal fit, Internal adaptation, CAD-CAM, Milling parameters, Fixed partial dentures

## Abstract

**Objective:**

To evaluate marginal fit and internal adaptation of three-unit Zr frameworks fabricated from four Zr CAD/CAM milling systems.

**Methods:**

Fixed partial denture models were replicated (40 stone models) using Polyvinyl Siloxane impression material (PVS) and type IV stone for Zr framework fabrication. FPDs were milled with four CAD/CAM systems, Group-II: LAVAL Zirconia milled by LAVA , Group-2: Vita In-Ceram YZ milled by Cerec®, Group-3: Zirconia milled by GM1000 and Group-4: Zirconia milled by DWX-50N. Twelve marginal gap measurements per framework were performed at pre-established points, with a metallurgical microscope (Zeiss, Germany) at 500X magnification. Eight measurements of cement space per section were performed for adaptation. Data was analyzed using ANOVA and Tukey post hoc test.

**Results:**

Zirconia FPD frameworks exhibited gaps ranging from 16 to 50.1 µm for marginal fit and 26.8 to 102.5 µm for internal adaptation. Group-3 [20.8 (8.3) µm & 50.3 (11.4) µm] and Group-4 [16.0 (4.0) µm & 40.2 (8.8) µm] specimens showed significantly lower marginal fit and internal adaptation gaps compared to Group-I [50.1 (13.4) µm & 100.5 (16.7) µm] and Group-2 [38.9 (8.2) µm & 102.5 (13.4) µm] specimens respectively.

**Conclusions:**

Different CAD-CAM systems for fabrication of Zr FPD frameworks displayed a significant influence on marginal fit and internal adaptation of restorations.

## INTRODUCTION

There has been a paradigm shift in the field of dentistry, especially with the growing awareness for high-quality cosmetic dentistry and biocompatibility.^1^ This inspired development of new dental ceramics for single crowns, fixed partial denture, and implant restorations to provide alternative treatment options. In addition to the advanced improvement in the mechanical properties of the dental ceramic materials, new sophisticated processing technologies and systems have been introduced for the production of all-ceramic restorations.^2^ Different constructing techniques used to fabricate ceramic restorations include, hot-pressing, slip-casting, and computer-aided design-computer aided manufacturing (CAD-CAM) systems.

CAD-CAM systems employ contemporary technologies for milling restorations from densely sintered or partially sintered ceramic blocks.^3^ A scanner digitizes the prepared tooth or die and the restoration is fabricated according to the previously established design. However, contemporary CAD-CAM systems do present shortcomings like the use of reflective powder coating before scanning of the tooth margins causing restorative distortion.^4^ In addition, CAD/CAM machining may lead to chipping defects, surface flaws and micro cracking due to the grinding processes. Such defects not only affect the fit of the restoration, but may also reduce its mechanical strength and clinical prognosis.^5^

One critical criterion for the success of CAD-CAM manufacturing systems is the marginal integrity and internal fit accuracy of the restorations. Accurate fit of restorations is extremely essential to achieve acceptable longevity.^6^ The presence of discrepancies at the crown margins favors plaque accumulation and microleakage, dissolution of cement, leading to secondary caries and periodontal disease.^6^ Multiple studies have assessed the marginal fit and adaptation of CAD-CAM restorations.^7^ It is suggested that the marginal accuracy of restorations made with CAD-CAM systems may be influenced by the designing software’s and parameters.^8^

It is reported that parameters specific to the milling and machining process including number and dimensions of burs, axis of milling and machining type (soft or hard) may effect the fit and adaptation of CAD-CAM restorations.^9^,^10^ A three or four axis machine may not be capable of producing undercuts, therefore show low accuracy and marginal fit.^11^ Furthermore, milling of partially stabilized zirconia in dry conditions has shown better restorative accuracy then wet milling.^10^ However, in a study by Kirsch et al, restoration fabricated with a 4-axis CEREC milling machine revealed comparable fit outcomes to restoration made with 5-axis milling machines.^12^ Therefore a controversy exists in these findings. It is hypothesized that partially stabilized zirconia restorations when fabricated using different CAD-CAM milling machines and systems will show a significant difference in their marginal fit and adaptation. Therefore, the aim of the study was to compare the marginal fit and internal adaptation of partially stabilized zirconia fixed partial dentures fabricated with different CAD-CAM systems.

## METHODS

This experimental study was performed over a period of twelve months and reported inline with the checklist for reporting in-vitro studies (CRIS). The project was approved by the ethics and research committee at specialist practice and research center. (Ref. FR-031 June 21^st^ 2019)

### Preparation of master model

**Fig.1:**
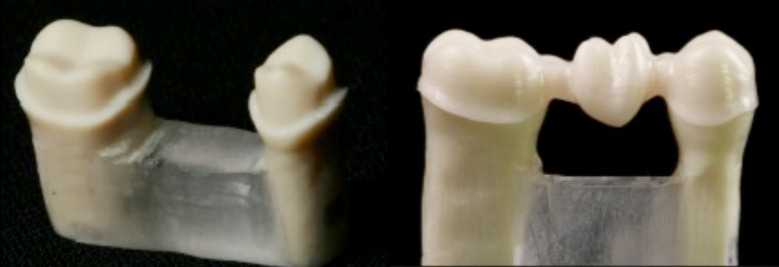
Prepared teeth model for scanning (A); Partially stabilized Zr milled framework (B).

Acrylic resin teeth (second premolar and second molar) were mounted in clear EpoxiCure® Epoxy Resin (Buehler, USA) with space for a missing first molar tooth to form the master model (Fig.1-A). The mounted teeth were prepared for all-ceramic zirconia fixed partial denture (FPDs) restorations followed preparation guidelines by Charles Goodacre et al. with features of deep chamfer finish lines, 2 mm functional cusp bevel, 1.5 mm axial surface and 10°-20° total occlusal convergence angle. A carbide-finishing bur was used to finish the entire preparation. The model teeth were cleaned using a steamer.

### Impression and preparation of master dies

Using polyvinyl siloxane impression material (Reprosil, DentsplySirona, MI, USA) in sectioned stock tray, the “master model” was impressed with a light body material at the finish line and heavy body material in the sectioned tray. After 5-6 minutes of setting, each impression was examined for defects or air bubbles. A total of 40 impressions were made.

All impressions were carefully boxed with boxing wax (Kerr). Thirty impressions were poured with type IV stone (Whip mix), generating thirty stone models for fixed partial denture (FPDs) frameworks fabrication. The other ten impressions were poured with scan able stone (Diamond die, HI-Tec) according to manufacture’s instructions (liquid/powder ratio, 19 to 20 cc water to 100 grams of powder). Powder was added gradually to water and vacuum mixed for 20 to 40 seconds. A total of 40 models were fabricated.

### Fabrication of zirconia frameworks

Partially stabilized Zirconia fixed partial denture (FPD’s) frameworks were milled for all models (forty) using four CAD-CAM systems. This resulted in four study groups based on the type of manufacturing techniques and systems (n=10). A total of forty zirconia frameworks were fabricated using the recommended materials and following the manufacturers instructions (Fig.1-B). The four systems used are as follows,

**Group-II:** LAVAG Zirconia (3M ESPE, US) frameworks milled by LAVA CNC 240.

**Group-2:** Vita In-Ceram YZ (VITA, Germany) frameworks, scanned with InEos Red Scan (Sirona Dental Systems, Germany), milled with Cerec® inLab (Sirona Dental Systems, Germany).

**Group-3:** AadvaG Zirconia (Zr) (GC Advanced technologies Inc.) frameworks milled by GM1000 (GC Advanced technologies Inc.).

**Group-4:** Katana Zirconia (Noritake®, Japan) frameworks milled by DWX-50N (Noritake®, Japan).

### Marginal fit measurements

**Fig.2:**
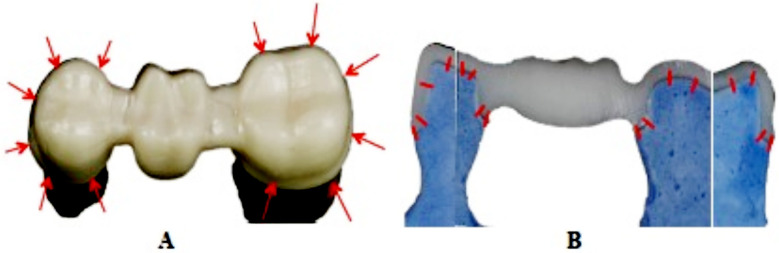
Point locations for measurements of marginal gap (A) and internal adaptation (B) of fixed partial denture frameworks.

Each framework was secured on the respective stone model with a special clamp with uniform force at the pontic area. A metallurgical microscope (Zeiss, Germany) connected to a high precision digital video-micrometer (Javelin JV6000, California, USA) was used as for marginal gap measurements at 500x magnification. A total of twelve marginal gap measurements per framework were performed at pre-established points, six (6) measurements per tooth “mesio-buccal, mid-buccal, disto-buccal, mesio-lingual, mid-lingual, disto-lingual” for both prepared abutment teeth (Fig.2-A). The images of the specimens measured were saved as bit-map (bmp) files on the Windows® based computer attached to the microscope. The specimens were positioned in away that the margins were vertical on the measuring panel allowing the vertical lines in the video-micrometer to measure the gap (Fig.2-A). The marginal fit and internal adaptation testing was performed by single operator (KAA)

### Internal fit for zirconia CAD/CAM frameworks

All frameworks were cemented on respective stone die using dual cure resin cement RelyX™ Unicem Aplicap™ (3M ESPE). Cementation was done individually and one RelyX™ Unicem Aplicap™ capsule was used per framework. The RelyX™ Unicem Aplicap™ was activated and triturated for 15 seconds (4,300 RPM) utilizing a 3M ESPE CapMix™ Triturator according to manufacturer’s instructions. The cement was dispensed into the framework and then placed on the die. A weight device axially loaded frameworks on the dies at 50N for 10 minutes with the metal ball centrally at the occlusal part of the pontic. Excess cement was cleaned and allowed to auto cure (eight minutes). All cemented frameworks were carefully boxed (boxing wax-Kerr) and embedded using clear epoxy resin (EpoxiCure® Epoxy Resin- Buehler, USA). Specimens were sectioned (Isomet® 2000 Precision diamond saw- Buehler, USA) under 200 grams load at a speed of 500 rpm under copious irrigation, mesio-distally and bucco-lingually through the center of abutment teeth, dividing each specimen six parts. (Fig.2-B). The finished and polished (Buehler grinding-polishing system-Buehler Ltd) specimens were assessed at eight pre-established measurements of cement space for both abutment teeth (bucco-lingual and mesio-distal). The specimens were secured to metal rectangular plates attached with grey modeling clay and placed in the metallurgical microscope (Zeiss, Germany) connected to a high precision digital video-micrometer (Javelin JV6000, California, USA). Measurements were made at 500x magnification with the flat surface of the specimen facing the optical lens. Eight pre-established measurements (Fig.2-B) of cement space per section were performed for both abutment teeth for both specimen sides mesio-distal and bucco-lingual giving 16 gap measurements for each framework. Resulting in a total of 160 readings being measured for each system (group). Both marginal fit and internal adaptation were expressed in microns (μm). Means and standard deviations among the study groups were compared employing ANOVA and Tukey Kramer multiple comparisons test. Some parts of the methodology were adopted from previous studies.^3^

## RESULTS

The maximum and minimum marginal fit gap was observed in Group-II (50.1 ± 13.4 µm) (LAVAm Zirconia- LAVA CNC 240) and group-4 (16.0 ± 4.0 µm) (Katana Zirconia-DWX-50N) specimens respectively (Table-I). However, the marginal fit gap among Group-2 (Vita In-Ceram YZ- Cerec® inLab) and group-3 (Aadva Zirconia-GM1000) specimens were 38.9 ± 8.2 µm and 20.8 ± 8.3 µm respectively. ANOVA showed a significant difference (p<0.05) in marginal gap of restorations among the tested study groups. Group-II specimen showed significantly higher (p <0.05) marginal gap (50.1 ± 13.4 µm) than all other groups. Specimens in group-2 showed significantly lower (p<0.05) marginal gap than Group-II and significantly higher (p<0.05) value compared to Groups three and four respectively (Table-I). Group-3 specimens showed lower (p<0.05) marginal gap than Groups one and two, however they showed higher (p<0.05) marginal gap than Group-4 specimens. Group-3 and Group-4 specimens showed comparable (p>0.05) outcomes for marginal gap of fixed partial denture frameworks.

**Table-I:** Comparison of means and standard deviations of Marginal Fit and internal adaptation (μm) among study groups.

Study Groups	Marginal Fit µm Mean (SD)	p-value*	Internal Adaptation (µm)	p-value*
Group-II	50.1 (13.4)^a^		100.5 (16.7)^a^	
Group-2	38.9 (8.2)^b^	< 0.05	102.5 (13.4) ^a^	<0.05
Group-3	20.8 (8.3)^c^		50.3 (11.4)^b^	
Group-4	16 (4.0)^c^		40.2 (8.8)^b^	

Dissimilar superscript alphabets denote statistical different (Tukey-Kramer Test)-

* ANOVA

The means and standard deviations for internal adaptation of frameworks in each group are presented in Table-I. The specimens in group-4 (Katana Zirconia-DWX-50N) showed the least gap (40.2 ± 8.8 µm) for internal adaptation, however Group-II (LAVAr Zirconia- LAVA CNC 240) (100.5 ± 16.7 µm) showed the highest adaptation gaps among all groups. The internal adaptation values for group-2 and group-3 specimens were 102.5 ± 13.4 µm and 50.3 ± 11.4 µm respectively. Internal adaptation gap among group-3 (50.3 ± 11.4 µm) and 4 (40.2 ± 8.8 µm) specimens were statistically comparable (p>0.05). However, internal adaptation gap among group-3 (50.3 ± 11.4 µm) and 4 (40.2 ± 8.8 µm) specimens was significantly less (p<0.05) compared to Group-II (100.5 ± 16.7 µm) and group-2 (102.5 ± 13.4 µm) specimens respectively. Internal adaptation gap was comparable among Group-II (100.5 ± 16.7 µm) and group-2 (102.5 ± 13.4 µm) specimens (p>0.05) (Table-I).

## DISCUSSION

The present study was based on the hypothesis, partially stabilized zirconia restorations when fabricated using different CAD-CAM milling systems will show a significant difference in their marginal fit and adaptation. It was observed that FPD fabricated with Katana Zirconia-DWX-50N (group-4) and AadvaD Zirconia-GM1000 (group-3) showed better marginal fit and internal adaptation compared to LAVA Zirconia- LAVA CNC 240 (Group-II) and Vita In-Ceram YZ- Cerec® inLab (group-2) specimens. Therefore the hypothesis was accepted. A myriad of reasons are implicated for the findings including material properties, milling parameters (3 or 5 axis, soft or hard machining, burs size) and cement space and viscosity.

Multiple factors involved in the methodology of the study can influence the marginal gap and internal adaptation, including, cement type, cement thickness, cement mixing, seating pressure and preparation margin type.^13^ A standard resin cement (RelyX™ Unicem) was auto-mixed and FPDs were seated at a standard load of 50N for 10 minutes for cementation. In addition, a deep chamfer margin was prepared for all specimens as it allows for thickness of ceramic and smooth flow and extrusion of luting cements. A single operator performed measurements of the fit and adaptation. Intra-examiner reliability was achieved after multiple measurements and a kappa score of 0.85 was achieved. Due to rapidly changing technology and materials for dental restorations and cements, there are no gold standards and guidelines for the acceptable clinical and biological fit and adaptation of indirect fixed partial dentures. The American Dental Association specification states that the luting cement film thickness for a crown restoration should be no more than 25 μm using a type 1 luting agent or 40 μm with a type II luting agent.^14^ Christensen (1966), in a classic study, reported that clinically detectable marginal discrepancy for sub-gingival margins is 34-119 μm, and 2-51 μm for supra-gingival margins.^15^ He also related, that marginal discrepancies of 39 μm or more in visually accessible surfaces are unacceptable.^15^ Some in vivo studies showed that clinically acceptable margins can range from 7-65 μm.^16^ However, gaps of less than 80 μm were proven to be very difficult to detect clinically,^17^ and several authors have considered that marginal discrepancies between 100-150 μm to be clinically acceptable.^18^

In the present study, AadvaA Zirconia (Zr) frameworks milled by GM1000 and Katana Zirconia frameworks milled by DWX-50N were compared to LAVA Zirconia frameworks milled by LAVAs CNC 240 and Vita In-Ceram YZ frameworks milled by Cerec® inLab. There is limited data reporting the use of Katana zirconia and AadvaA Zirconia in the literature.^19^ It was observed that the marginal gap widths for all tested materials and systems appeared to be considerably lower than what is reported in previous studies.^20^ In the present study, Vita In-Ceram YZ frameworks showed mean marginal gaps of 38.9±8.2 µm. In a similar study by Att et al (2009), mean marginal gap was 64 µm for VITA In-Ceram YZ, which is considerably higher.^20^ In addition in the present study, group-4 (Katana Zirconia-DWX-50N) specimens showed lower marginal gap compared to specimens in Group-II (LAVAL CNC 240). Katana Zirconia-DWX-50N milling machine is 5-axis (x, y, z, a, b) automatic tool changer (ATC) equipped with five milling tools capable of producing various shapes of zirconia restorations with undercuts. Its versatile 5th axis allows materials to tilt as much as ±20 degrees.^21^ By contrast, Lava CNC 240 is a computer-controlled precision milling machine with three linear axes (X, Y, Z) and a rotational axis (A). Therefore a difference in milling axis may influence the milling accuracy. In addition, the type and number of burs employed in different milling systems may influence the precision milling ability of the systems resulting is compromise in marginal fit outcomes.

The present study revealed, that the internal adaptation of fixed partial denture specimens was significantly different based on the type of CAD-CAM system used. Specimens milled in group-3 (Aadva Zirconia GM1000) and group-4 (Katana Zirconia-DWX-50N) showed significantly lower internal adaptation gaps than Group-II (LAVAL CNC 240) specimens. A possible explanation of these findings can be derived from the fact that LAVA Zr milling undergoes post milling sintering for milling (soft machining) efficiency of Zr substructure, resulting in a linear shrinkage of nearly 20%.^22^ However there are compensatory mechanisms in the software and scanning process, sintering is a possible cause for increased discrepancies in the marginal fit and internal adaptation of zirconia frameworks, as shown in the present study. A systematic review by Abduo et al, concluded that Zr framework fit discrepancies are related to soft machining, curvatures and long framework spans.^23^ By contrast specimens in group-3 and group-4 are milled from fully sintered Zr blocks. Moreover, the specimens in group-3 were fabricated with GM 1000, a 5-axis laser milling system. It is suggested previously, that a 3-axis milling system (LAVA CNC 240) shows insufficient accuracy of longitudinal milling in comparison to 5-axis laser milling, therefore resulting in higher internal adaptation gaps for Zr frameworks.^19^ These findings of better fit and adaptation of Zr frameworks made from 5-axis laser milling compared to a 3 to 4 axis milling are in line with a previous study.^19^ Therefore a 5-axis laser milling system for post sintered Zr is recommended for frameworks on abutments of complex anatomy and tall height with teeth and dental implants. Furthermore, other factors such as bur size, dry and wet milling, cement space and operator experience are some factors which can be implicated for the observations in the present study.^23^-^24^

### Limitations of the study

Outcomes of the present study should be interpreted in light of the limitations. The Zr materials used in the study groups were manufactured by different companies, therefore may differ in composition. In addition, FPD frameworks were seated on artificial teeth abutments with a static force of 50N, however intraoral forces are dynamic and can be upto 400N in some cases. The cemented specimens were sectioned using a destructive method, which may have possibly influenced the fit and adaptation measurement outcomes. Hard machining with a 5-axis milling although showed better fit and adaptation outcomes for Zr frameworks in the present study, however there is a continuous need for further improvements in the technology of hard machining with regards to cutting efficiency, time consumption and surface properties of milled materials. In addition, the introduction of additive technology (3D Printing) for the manufacture of metal and Zr frameworks of dental restorations provide improvement potential in restorative fit and adaptation. Therefore, further investigations on the properties of multiple axis, laser milled and printed, hard machined Zr framework including surface topography, flexural strength, fracture toughness and color stability are recommended.

## CONCLUSION

The Zr CAD-CAM systems assessed exhibited marginal fit and internal adaptations measurements of 16.0 to 50.1 µm and 40.2 to 102.5 µm respectively. For both fit and adaptation, specimens in Group-3 (Aadvam Zirconia (Zr)- GM1000) and Group-4 (Katana Zirconia- DWX-50N) showed better outcomes then specimens in Group-II (LAVA Zirconia/ LAVA/ CNC 240) and group-2 (Vita In-Ceram YZ/ Cerec® inLab). Marginal fit and internal adaptation values of all materials and systems tested were within clinically acceptable standards.

### Authors’ Contribution:

**KA:** Experimentation, Data collection, study design, manuscript writing, final manuscript approval.

**AM:** Data collection, study design, manuscript drafting, data analysis, funding and equipment, manuscript approval.

**RA:** Specimen design and preparation, Data collection, manuscript approval and data interpretation.

**FV & TA:** Data collection, sample preparation, writing, revision, editing, final manuscript approval, table and figure designing.

As per the ICMJE authorship requirement, all authors are responsible and accountable for the accuracy or integrity of the work.
